# Cutaneous Toxicity Associated With Enfortumab Vedotin: A Real-Word Study Leveraging U.S. Food and Drug Administration Adverse Event Reporting System

**DOI:** 10.3389/fonc.2021.801199

**Published:** 2022-01-19

**Authors:** Hui Yang, Xiaojia Yu, Zhuoling An

**Affiliations:** Department of Pharmacy, Beijing Chao-Yang Hospital, Capital Medical University, Beijing, China

**Keywords:** cutaneous toxicity, EV, Food and Drug Administration Adverse Event Reporting System, disproportionality analysis, real-word study

## Abstract

**Introduction:**

Enfortumab vedotin (EV) has been demonstrated to have a significant response rate in early phase trials and is known for its tolerable side-effect profile. Emerging case reports have raised awareness of cutaneous toxicities, which may be a potentially fatal complication.

**Objective:**

To assess the potential relevance between EV and cutaneous toxicities reports through data mining of the U.S. Food and Drug Administration (FDA) adverse event reporting system (FAERS).

**Methods:**

Data from January 1, 2019, to November 4, 2021, in the FAERS database were retrieved. Information component (IC) and reporting odds ratio (ROR) were used to evaluate the association between EV and cutaneous toxicities events.

**Results:**

EV was significantly associated with cutaneous toxicities in the database compared with both all other drugs (ROR 12.90 [10.62–15.66], IC 2.76 [2.52–3.01], middle signal) and platinum-based therapy (ROR 15.11 [12.43–18.37], IC 2.91 [2.66–3.15], middle signal) in the FAERS database. A significant association was detected between EV and all the cutaneous adverse effects (AEs) except erythema, palmar–plantar erythrodysesthesia syndrome, and dermatitis allergic. Both Stevens–Johnson syndrome and toxic epidermal necrolysis occurred 15 times as frequently for EV compared with all other drugs (ROR = 15.20; ROR = 15.52), while Stevens–Johnson syndrome occurred 18 times and toxic epidermal necrolysis occurred 7 times as frequently for EV compared with platinum-based therapy in the database (ROR = 18.74; ROR = 7.80). All groups that limited the gender and age showed a significant association between EV and cutaneous toxicities.

**Conclusions:**

A significant signal was detected between EV use and cutaneous toxicities. It is worth noting that Stevens–Johnson syndrome and toxic epidermal necrolysis were significantly associated with EV use.

## Introduction

Urothelial cancer (UC) is the ninth most common cancer worldwide (1). At presentation, about 70% of patients have non-muscle-invasive disease and 25% muscle-invasive disease, and 5% will be metastatic (2). Early stages of disease (non-muscle-invasive UC and muscle-invasive disease UC) are often treated with cisplatin-based chemotherapy with objective response rates of approximately 50% (3). And the immune checkpoint inhibitor (ICI) is considered the standard of care in patients who are either cisplatin-unfit or platinum-refractory (4). However, patients with metastatic UC (mUC) with disease progression on both platinum-based chemotherapy and an ICI had few treatment options available and often have a dismal prognosis (5).

Enfortumab vedotin (EV) is an antimitotic antibody–drug conjugate (ADC) that inhibits microtubule assembly, which received Food and Drug Administration (FDA)-accelerated approval for the treatment of adult patients with locally advanced or mUC who had failed in the previous treatment of ICIs and platinum-based chemotherapeutic agents in 2019 (6). The drug has been demonstrated to have a significant response rate in early phase trials and is known for its tolerable side-effect profile (7–11). Common toxicities that have been attributed to EV were fatigue, peripheral neuropathy, skin rashes, gastrointestinal issues, and hematological suppression (12). The first case of cutaneous toxicities induced by EV was found in 2019 (13). Recently, emerging case reports have raised awareness of cutaneous toxicities, which may be a potentially fatal complication (14–17). But the precise descriptions of cutaneous toxicities were limited. Perhaps because of inadequate understanding as a form of EV-related cutaneous toxicities, data are derived primarily from case reports and clinical trials that may not correctly represent the real world. Moreover, the characteristics, outcomes, and types of EV-related cutaneous toxicities are still unknown.

Considering the wide clinical use of EV and the potentially fatal consequences of EV-associated cutaneous toxicities, it is important to identify its clinical manifestations. Therefore, we aim to assess the potential relevance between EV and cutaneous toxicities through data mining of the U.S. FDA adverse event (AE) reporting system (FAERS).

## Materials and Methods

### Data Sources and Study Variables

The data were obtained from the FAERS database, which is publicly available and contains spontaneous AE reports submitted to the U.S. FDA by healthcare professionals, consumers, drug manufacturers, and others. The FAERS database Quarterly Data Files (January 1, 2019, to November 4, 2021) were used. OpenVigil FDA, a validated pharmacovigilance tool, was adapted to access the FDA drug-event database with the additional openFDA drug mapping and duplicate detection functionality (18–20).

### Pharmacovigilance Study Procedures

The reports in the FAERS database were coded using preferred terms (PTs) from the Medical Dictionary for Regulatory Activities. After literature review and summary of previous studies, we considered the following PTs as related to cutaneous toxicities: rash [10037844], rash pruritus [10037884], pruritus [10037087], rash erythematous [10037855], Stevens–Johnson syndrome [10042033], dry skin [10013786], toxic epidermal necrolysis [10044223], skin exfoliation [10040844], dermatitis bullous [10012441], rash maculopapular [10025423], skin discoloration [10040829], erythema [10015150], rash papular [10037876], skin reaction [10040914], skin toxicity [10059516], symmetrical drug-related intertriginous and flexural exanthema [10078325], dermatitis allergic [10012434], exfoliative rash [10064579], palmar–plantar erythrodysesthesia syndrome [10033553], and rash macular [10037867]. The clinical characteristics (gender, age, reporting time, etc.) of patients were collected.

### Statistical Analysis

Standard descriptive statistics were used to summarize the study population characteristics. We conducted a disproportionality analysis using the Bayesian confidence propagation neural network of information component (IC) and reporting odds ratio (ROR) to calculate disproportionality (21). ROR and IC are recognized disproportionality methods to identify whether a given AE (in this case, cutaneous toxicities) is reported more frequently than expected with a given drug (in this case, EV), which allows testing the possible disproportionate association between a drug and an AE (18). For IC, a significant signal was defined as the lower bound of the 95% CI (IC_025_) exceeded 0. If 0 < IC_025_ ≤ 1.5, then it is considered as weak signal; if 1.5 < IC_025_ ≤ 3.0, then it is considered as middle signal; if IC_025_ > 3.0, then it is considered as strong signal (22). Since IC-based signals were included in ROR-based ones (23), ROR was also calculated, and the significant signal was defined as the lower bound of the 95% CI (ROR_025_) exceeded 1, with at least 3 cases (24–26). All the analyses were performed using R version 3.2.5. The IC and ROR with 95% CI can be calculated by the following:


$$\text{IC}=\log_{2}\frac{\left(cxy+\gamma \text{xy)(}C+\alpha \text{)(}C+\beta \right)}{\left(C+\gamma )(\text{cx+}\alpha \text{x})(\text{cy}+\beta \text{y}\right)}=\log_{2}\frac{(cxy+\gamma \text{xy)}\gamma }{\left(C+\gamma \right)}$$

$$SD=\sqrt{\frac{\left\{\left(\frac{C-cxy+\gamma -\gamma \text{xy}}{\left(cxy+\gamma \text{xy})(1+C+\gamma \right)}\right)+\left(\frac{C-cx+\alpha -\alpha \text{x}}{\left(cx+\alpha \text{x})(1+C+\alpha \right)}\right)+\left(\frac{C-cy+\beta -\beta y}{\left(cy+\beta \text{y})(1+C+\beta \right)}\right)\right\}}{{\left(ln2\right)}^{2}}}$$

$${\text{IC}}_{95\%\text{CI}}=\text{IC}\pm 2\text{SD}$$

$$\text{ROR}=\frac{\left(\text{a/c}\right)}{\left(\text{b/d}\right)}=\frac{\text{ad}}{\text{bc}}{\text{ ROR}}_{95\%\text{CI}}=\text{e}\ln(ROR)\pm 1.96\sqrt{\left(\frac{1}{a}+\frac{1}{b}+\frac{1}{c}+\frac{1}{d}\right)}$$

a = number of target AE of EV alone

b = number of other AEs of EV alone

c = number of target AE of other drugs except for EV

d = number of other AEs of other drugs except for EV


$${\text{c}}_{\text{xy}}=\text{a},{\text{ c}}_{\text{x}}=\text{a}+\text{b},{\text{ c}}_{\text{y}}=\text{a}+\text{c},\text{ C}=\text{a}+\text{b}+\text{c}+\text{d},\;\gamma_{\text{xy}}=1,\;\alpha =2,\;\beta =2,\;\alpha_{x}=1,\;\beta_{\text{y}}=1$$

$$\gamma =\gamma_{\text{xy}}\frac{\left(C+\alpha )(C+\beta \right)}{\left(\text{cx+}\alpha \text{x})(\text{cy}+\beta \text{y}\right)}$$

## Results

### Descriptive Analysis

Overall, 409 AE reports related to EV and 212 AE reports related to cutaneous toxicities were submitted to the FAERS between January 1, 2004, and November 4, 2021. We screened all reported EV-related cutaneous toxicities, and the clinical characteristics are summarized in **Table 1**. Rash was the most common cutaneous toxicities related to EV. All the cases were reported between 2020 and 2021. Most cases were male (76.42%). The median age of cases was 74.5 (6–92) years. Most cases were EV monotherapy (83.49%), while only a few patients accepted combination therapy (**Table 1**).

**Table 1:** Characteristics of patients with enfortumab vedotin associated cutaneous toxicities sourced from the FAERS database.

Characteristics	N. of case	Gender			Age					
		Male n (%)	Female n (%)	Unknown or missing n (%)	Median (IQR)	≤60 n (%)	61–70 n (%)	71–80 n (%)	≧81 n (%)	Unknown or missing n (%)
**Total**	212	162 (76.42)	42 (19.81)	8 (3.77)	74.5 (6–92)	19 (8.96)	33 (15.57)	45 (21.23)	15 (7.08)	100 (47.17)
**EV monotherapy**	177	137 (77.40)	35 (19.77)	5 (2.83)	73 (6–92)	16 (9.04)	28 (15.82)	32 (18.08)	14 (7.91)	87 (49.15)
**Combination therapy**										
**EV + pembrolizumab**	22	19 (86.36)	1 (4.55)	2 (9.09)	72 (60–78)	2 (9.09)	5 (22.73)	12 (54.55)	0 (0.00)	3 (13.64)
**EV + atezolizumab**	2	1 (50.00)	1 (50.00)	0 (0.00)	—	0 (0.00)	0 (0.00)	1 (50.00)	0 (0.00)	1 (50.00)
**EV + cisplatinum**	3	1 (33.33)	2 (66.67)	0 (0.00)	—	0 (0.00)	0 (0.00)	0 (0.00)	0 (0.00)	3 (100.00)
**EV + carboplatin**	2	1 (50.00)	0 (0.00)	1 (50.00)	—	0 (0.00)	0 (0.00)	0 (0.00)	1 (50.00)	1 (50.00)
**EV + pembrolizumab + erdafitinib**	1	1 (100.00)	0 (0.00)	0 (0.00)	—	1 (100.00)	0 (0.00)	0 (0.00)	0 (0.00)	0 (0.00)
**EV + pembrolizumab + erdafitinib**	3	2 (66.67)	1 (33.33)	0 (0.00)	—	0 (0.00)	0 (0.00)	0 (0.00)	0 (0.00)	3 (100.00)
**EV + pembrolizumab + cisplatinum**	2	0 (0.00)	2 (100.00)	0 (0.00)	—	0 (0.00)	0 (0.00)	0 (0.00)	0 (0.00)	2 (100.00)
**Adverse Effects (AEs)**										
**Rash**	79	58 (73.42)	19 (24.05)	2 (2.53)	74 (6–90)	8 (10.13)	7 (8.86)	10 (12.66)	8 (10.13)	46 (58.23)
**Rash pruritus**	16	10 (62.50)	6 (37.50)	0 (0.00)	71.5 (8–92)	3 (18.75)	2 (12.50)	5 (31.25)	2 (12.50)	4 (25.00)
**Pruritus**	14	13 (92.85)	1 (7.14)	0 (0.00)	72 (65–88)	0 (0.00)	2 (14.29)	3 (21.43)	1 (7.14)	8 (57.14)
**Rash erythematous**	13	12 (92.31)	1 (7.69)	0 (0.00)	73 (7–81)	2 (15.38)	1 (7.69)	5 (38.46)	1 (7.69)	4 (30.77)
**Stevens–Johnson syndrome**	13	9 (69.23)	2 (15.38)	2 (15.38)	76 (67–78)	0 (0.00)	1 (7.69)	5 (38.46)	0 (0.00)	7 (53.85)
**Dry skin**	12	9 (75.00)	3 (25.00)	0 (0.00)	65.5 (40–83)	1 (8.33)	2 (16.67)	0 (0.00)	1 (8.33)	8 (66.67)
**Rash maculopapular**	10	8 (80.00)	2 (20.00)	0 (0.00)	70 (60–92)	1 (10.00)	4 (40.00)	2 (20.00)	1 (10.00)	2 (20.00)
**Toxic epidermal necrolysis**	9	5 (55.56)	1 (11.11)	3 (33.33)	72 (67–78)	0 (0.00)	1 (11.11)	3 (33.33)	0 (0.00)	5 (55.56)
**Skin exfoliation**	8	6 (75.00)	2 (25.00)	0 (0.00)	67 (40–85)	1 (12.50)	4 (50.00)	1 (14.29)	1 (12.50)	1 (12.50)
**Dermatitis bullous**	6	6 (100.00)	0 (0.00)	0 (0.00)	77 (65–78)	0 (0.00)	1 (16.67)	3 (50.00)	0 (0.00)	2 (33.33)
**Skin discoloration**	6	5 (83.33)	1 (16.67)	0 (0.00)	65 (60–66)	1 (16.67)	3 (50.00)	1 (16.67)	0 (0.00)	1 (16.67)
**Blister**	5	4 (80.00)	1 (20.00)	0 (0.00)	72 (67–77)	0 (0.00)	1 (20.00)	1 (20.00)	0 (0.00)	3 (60.00)
**Erythema**	4	3 (75.00)	1 (25.00)	0 (0.00)	60 (60–69)	2 (50.00)	1 (25.00)	0 (0.00)	0 (0.00)	1 (25.00)
**Skin reaction**	4	3 (75.00)	1 (25.00)	0 (0.00)	—	0 (0.00)	0 (0.00)	0 (0.00)	0 (0.00)	4 (100.00)
**Exfoliative rash**	3	3 (100.00)	0 (0.00)	0 (0.00)	75 (71–76)	0 (0.00)	0 (0.00)	3 (100.00)	0 (0.00)	0 (0.00)
**Palmar–plantar erythrodysesthesia syndrome**	3	2 (75.00)	1 (25.00)	0 (0.00)	—	0 (0.00)	0 (0.00)	1 (33.33)	0 (0.00)	2 (66.67)
**Skin toxicity**	3	2 (66.67)	0 (0.00)	1 (33.33)	—	0 (0.00)	1 (33.33)	0 (0.00)	0 (0.00)	2 (66.67)
**Symmetrical drug-related intertriginous and flexural exanthema**	3	3 (100.00)	0 (0.00)	0 (0.00)	70 (70–81)	0 (0.00)	2 (66.67)	1 (33.33)	0 (0.00)	0 (0.00)
**Dermatitis allergic**	1	1 (100.00)	0 (0.00)	0 (0.00)	—	0 (0.00)	0 (0.00)	1 (100.00)	0 (0.00)	0 (0.00)

N, number; EV, enfortumab vedotin; FAERS, Food and Drug Administration (FDA) adverse event reporting system; IQR, interquartile range.

### Signal Values Associated With Enfortumab Vedotin

EV was significantly associated with cutaneous toxicities compared with both all other drugs (ROR 12.90 [10.62–15.66], IC 2.76 [2.52–3.01], middle signal, **Table 2**) and platinum-based therapy (ROR 15.11 [12.43–18.37], IC 2.91 [2.66–3.15], middle signal, **Table 2**). And significant association was detected between EV and all the cutaneous AEs except erythema, palmar–plantar erythrodysesthesia syndrome, and dermatitis allergic (**Table 2**). Nine AEs were detected as middle signal including rash (IC_025_ = 2.85), rash erythematous (IC_025_ = 2.49), Stevens–Johnson syndrome (IC_025_ = 2.96), dry skin (IC_025_ = 2.15), rash maculopapular (IC_025_ = 1.51), toxic epidermal necrolysis (IC_025_ = 2.02), skin exfoliation (IC_025_ = 1.57), dermatitis bullous (IC_025_ = 1.91), and blister (IC_025_ = 1.97) compared with platinum-based therapy in the database, while rash pruritus was detected as strong signal (IC_025_ = 3.32).

**Table 2:** Disproportionality analysis of enfortumab vedotin and cutaneous toxicities.

Category	N. of case	ROR (ROR_025_–ROR_975_)		IC (IC_025_–IC_975_, signal strength)	
		Compared with all other drugs	Compared with platinum-based therapy	Compared with all other drugs	Compared with platinum-based therapy
**Cutaneous toxicities**	212	12.90 (10.62–15.66)	15.11 (12.43–18.37)	2.76 (2.52 to 3.01, middle)	2.91 (2.66 to 3.15, middle)
**AEs**					
**Rash**	79	11.64 (9.11–14.88)	12.29 (9.58–15.76)	3.18 (2.82 to 3.53, middle)	3.21 (2.85 to 3.56, middle)
**Rash pruritus**	16	15.91 (9.65–26.23)	41.21 (24.22–70.11)	3.74 (2.64 to 4.11, middle)	4.07 (3.32 to 4.82, Strong)
**Pruritus**	14	2.17 (1.27–3.69)	3.56 (2.09–6.09)	0.98 (0.20 to 1.77, weak)	1.62 (0.83 to 2.41, weak)
**Rash erythematous**	13	15.96 (9.19–27.74)	17.49 (9.91–30.86)	3.28 (2.47 to 4.09, middle)	3.31 (2.49 to 4.14, middle)
**Stevens–Johnson syndrome**	13	26.41 (15.20–45.90)	33.51 (18.74–59.94)	3.69 (2.88 to 4.50, middle)	3.79 (2.96 to 4.61, middle)
**Dry skin**	12	4.50 (2.81–8.88)	12.83 (7.14–23.05)	2.02 (1.18 to 2.86, weak)	3.00 (2.15 to 3.86, middle)
**Rash maculopapular**	10	8.74 (4.67–16.38)	7.74 (4.10–14.61)	2.58 (1.66 to 3.50, middle)	2.44 (1.51 to 3.37, middle)
**Toxic epidermal necrolysis**	9	30.05 (15.52–58.20)	15.34 (7.80–30.21)	3.48 (2.52 to 4.45, middle)	3.00 (2.02 to 3.98, middle)
**Skin exfoliation**	8	5.14 (2.55–10.34)	10.22 (5.02–20.81)	1.94 (0.92 to 2.97, weak)	2.6 (1.57 to 3.64, middle)
**Dermatitis bullous**	6	40.41 (18.04–90.53)	33.57 (14.35–78.53)	3.21 (2.02 to 4.39, middle)	3.10 (1.91 to 4.30, middle
**Skin discoloration**	6	6.34 (2.83–14.20)	14.00 (6.14–31.94)	2.04 (0.86 to 3.23, weak)	2.66 (1.47 to 3.85, middle)
**Blister**	5	4.72 (1.95–11.40)	—*	1.68 (0.38 to 2.97, weak)	3.28 (1.97 to 4.59, middle)
**Erythema**	4	0.93 (0.35–2.50)	0.70 (0.26–1.88)	−0.25 (−1.70 to 1.19, no)	−0.62 (−2.06 to 0.83, no)
**Skin reaction**	4	14.06 (5.25–37.66)	7.37 (2.72–19.97)	2.35 (0.91 to 3.79, weak)	1.92 (0.47 to 3.38, weak)
**Skin toxicity**	3	33.37 (10.71–103.94)	3.39 (1.08–10.62)	2.35 (0.69 to 4.01, weak)	1.11 (−0.56 to 2.78, no)
**Symmetrical drug-related intertriginous and flexural exanthema**	3	356.28 (113.77–1115.71)	—*	2.57 (0.91 to 4.23, weak)	2.56 (0.88 to 4.23, weak)
**Exfoliative rash**	3	55.28 (17.74–172.29)	27.60 (8.42–90.49)	2.44 (0.79 to 4.10, weak)	2.29 (0.61 to 3.96, weak)
**Palmar–plantar erythrodysesthesia syndrome**	3	6.53 (2.10–20.33)	1.44 (0.46–4.48)	1.65 (−0.014 to 3.31, no)	0.22 (−1.45 to 0.88, no)
**Dermatitis allergic**	1	—*	—*	−4.62 (−7.41 to −1.83, no)	0.63 (−2.17 to 3.42, no)

N, number; ROR, reporting odds ratio; ROR_025_, the lower end of the 95% confidence interval of ROR; ROR_975_, the upper end of the 95% confidence interval of ROR; IC, information component; IC_025_, the lower end of the 95% confidence interval of IC; IC_975_, the upper end of the 95% confidence interval of IC.

^*^ROR was not calculated for the reason that the cases were less than 3.

### Analysis of Life-Threatening Adverse Events Associated With Enfortumab Vedotin

Stevens–Johnson syndrome and toxic epidermal necrolysis were the life-threatening AEs induced by EV. Those two AEs were all detected as middle signal and significantly associated with EV use. Both Stevens–Johnson syndrome and toxic epidermal necrolysis occurred 15 times as frequently for EV compared with all other drugs in the database (ROR = 15.20 and ROR = 15.52), while Stevens–Johnson syndrome occurred 18 times and toxic epidermal necrolysis occurred 7 times as frequently for EV compared with platinum-based therapy in the database (ROR = 18.74; ROR = 7.80).

Thirty-five death cases from all causes related to EV were submitted to the FAERS, and three cases were reported to be related to cutaneous toxicities (8.57%). It is worth noting that three cases were all related to Stevens–Johnson syndrome. The mortality rate of Stevens–Johnson syndrome related to EV was 13.64% in the FAERS.

### Signal Values Associated With Different Groups of Cases

We analyzed the association between EV and cutaneous toxicities in different groups that limited the gender and age. All groups showed significant association. Significant middle signals of cutaneous toxicities were shown in all groups (**Table 3**).

**Table 3:** Disproportionality analysis of enfortumab vedotin and cutaneous toxicities in different groups of cases.

Category	N. of case	ROR (ROR_025_–ROR_975_)		IC (IC_025_–IC_975_, signal strength)	
		Compared with all other drugs	Compared with platinum-based therapy	Compared with all other drugs	Compared with platinum-based therapy
**Total**	212	12.90 (10.62–15.66)	15.11 (12.43–18.37)	2.76 (2.52–3.01, middle)	2.91 (2.66–3.15, middle)
**Gender**					
**Male**	162	13.12 (10.50–16.40)	15.11 (12.08–18.91)	2.73 (2.46–3.01, middle)	2.90 (2.62–3.18, middle)
**Female**	42	11.71 (7.65–17.91)	13.48 (8.81–20.64)	3.58 (2.05–3.12, middle)	2.75 (2.21–3.29, middle)
**Age**					
**≤60**	19	9.49 (5.20–17.32)	10.93 (5.98–10.96)	2.33 (1.55–3.11, middle)	2.49 (1.71–3.27, middle)
**61–70**	33	26.37 (14.32–48.55)	30.37 (16.49–55.94)	2.99 (2.35–3.63, middle)	3.15 (2.51–3.79, middle)
**71–80**	45	38.53 (21.15–70.19)	44.37 (24.35–80.87)	3.17 (2.60–3.72, middle)	3.33 (2.77–3.89, middle)
**≥81**	15	14.98 (7.01–32.01)	17.26 (8.07–36.88)	2.56 (1.66–3.46, middle)	2.71 (1.82–3.61, middle)

N, number; ROR, reporting odds ratio; ROR_025_, the lower end of the 95% confidence interval of ROR; ROR_975_, the upper end of the 95% confidence interval of ROR; IC, information component; IC_025_, the lower end of the 95% confidence interval of IC; IC_975_, the upper end of the 95% confidence interval of IC.

## Discussion

To our knowledge, this is the first comprehensive pharmacovigilance study on cutaneous toxicities associated with EV based on the FAERS database. Our study included the largest such collection of cases to date, and 212 AE reports related to cutaneous toxicities were analyzed.

Our study detected a significant signal between EV use and cutaneous toxicities. The most well-recognized AE of EV is rash. The rate of rash was noted in 48% of patients in the previous clinical trial (8). The median time to onset of skin reactions has been estimated to be 1 month. Of patients who experienced rash, nearly two-thirds experienced complete resolution, and approximately one-fifth experienced partial improvement (27). Besides rash, our study detected other cutaneous AEs induced by EV including pruritus and Stevens–Johnson syndrome. The mechanism for the AEs is unclear now. EV is an ADC with a monomethyl auristatin E (MMAE) payload targeting Nectin-4, a protein widely expressed on UC cells (28). Nectin-4 is important in the skin, which has a role in cell–cell adhesion, and a functional disturbance could lead to impaired cell–cell attachment (29, 30). Besides that, cutaneous toxicities also appeared to be a common AE in studies involving other ADC that incorporate MMAE (31–33). Therefore, dermatologic sequelae observed could be attributed solely to the MMAE payload. Alternatively, the proposed mechanism is targeting Nectin-4 by EV with the delivery of the MMAE payload to the skin resulting in the observed keratinocyte apoptosis (16).

Stevens–Johnson syndrome and toxic epidermal necrolysis were the life-threatening AEs. Those two AEs have always been not a recognized side effect of EV. The first case report of a 71-year-old male who suffered from EV-induced toxic epidermal necrolysis was published in 2020 (15). And Viscuse et al. highlighted a case of Stevens–Johnson syndrome/toxic epidermal necrolysis following enfortumab infusions in 2021 (16). Unfortunately, both of the patients in these cases were dead after treatment. Those cases aroused our attention on EV-induced life-threatening cutaneous toxicity. Our study found that those two AEs were significantly associated with EV use. This reminded doctors that patients must be monitored for cutaneous toxicities with early involvement of dermatology.

Our study found a significant signal of cutaneous toxicities in all groups that limited the gender and age. All the groups were detected as middle signal. Young people (≤60 years old) had slightly lower reporting frequencies for cutaneous toxicities compared with old people.

Our study has limitations. First, the FAERS database was a spontaneous reporting system. Underreporting, selective reporting, and many missing data could bring reporting bias. Second, the limited data might not contribute to a better comprehensive evaluation of EV-induced cutaneous toxicities. Third, disproportionality analysis is a suitable tool to quantitate signals for the AE. But the causal relationship between drugs (EV) and the AE (cutaneous toxicities) cannot be verified without a clinically performed causality assessment, while confounders such as comorbidity and concomitant drugs cannot also be assessed properly.

## Conclusion

Our study detected a significant signal between EV use and cutaneous toxicities. It is worth noting that Stevens–Johnson syndrome and toxic epidermal necrolysis were significantly associated with EV use. Patients must be monitored for cutaneous toxicities with early involvement of dermatology. Further study is required with better data sources and research design to draw conclusions on the strength of the relationships.

## Data Availability Statement

The datasets presented in this study can be found in FAERs database. Further inquiries can be directed to the corresponding authors.

## Author Contributions

HY was responsible for the study conception and design, data acquisition, data analysis and interpretation, manuscript preparation, and manuscript editing. XY was responsible for the data acquisition. ZA was responsible for the data analysis and interpretation. All authors contributed to the article and approved the submitted version.

## Conflict of Interest

The authors declare that the research was conducted in the absence of any commercial or financial relationships that could be construed as a potential conflict of interest.

## Publisher’s Note

All claims expressed in this article are solely those of the authors and do not necessarily represent those of their affiliated organizations, or those of the publisher, the editors and the reviewers. Any product that may be evaluated in this article, or claim that may be made by its manufacturer, is not guaranteed or endorsed by the publisher.

## Abbreviations

UC, urothelial cancer; ICI, immune checkpoint inhibitor; mUC, metastatic UC; EV, enfortumab vedotin; ADC, antibody–drug conjugate; FDA, Food and Drug Administration; FAERS, FDA adverse event reporting system; AEs, adverse events; PTs, preferred terms; IC, information component; ROR, reporting odds ratio; MMAE, monomethyl auristatin E.
